# Hysteresis and Synergy of the Central Commands to Muscles Participating in Parafrontal Upper Limb Movements

**DOI:** 10.3389/fphys.2019.01441

**Published:** 2019-11-26

**Authors:** Alexander I. Kostyukov, Oleksii V. Lehedza, Andrii V. Gorkovenko, Tetiana I. Abramovych, Wieslawa Pilewska, Viktor S. Mischenko, Mariusz Zasada

**Affiliations:** ^1^Department of Movements Physiology, Bogomoletz Institute of Physiology, National Academy of Sciences of Ukraine, Kyiv, Ukraine; ^2^Faculty of Physical Education, Health and Tourism, Institute of Physical Culture, Kazimierz Wielki University in Bydgoszcz, Bydgoszcz, Poland; ^3^Department of Physical Education, Gdansk University of Physical Education and Sport, Gdańsk, Poland

**Keywords:** motor control, muscles, electromyography, two-joint movements, muscle synergy

## Abstract

The averaged electromyograms (EMGs) were registered from the arm muscles of ten subjects in movements of the right hand performed under visual guidance on the horizontal plane along linear trajectories going parallel to the frontal plane at various distances from the trunk. The tests consisted of the steady movements (speed 4 cm/s) between two points symmetrical about the shoulder axis; the hand moved firstly from left to right, then in the opposite direction. The tests repeated ten times for each of two equal loads (10.2 N) applied to the hand along movement trajectory in the right- (*F*_*r*_) or leftward (*F*_*l*_) directions. The elbow and shoulder flexors reacted predominantly on *F*_*r*_ loads; the extensors were mostly activated by *F*_*l*_ loads. Positional changes of the averaged EMGs in both flexor and extensor muscles belonging to different joints demonstrated hysteresis properties; the respective hysteresis loops had counterclockwise direction in flexors and clockwise in extensors. The muscles predominantly opposing the loading forces of a given direction participate in a cocontraction mode as antagonists when the direction of load is changed; in this case, together with a decrease in the amplitude of the hysteresis loops, their direction is also reversed. The multiplication index of synergy (MIS), which is based on multiplication of the respective normalized averaged EMG records, has been proposed to evaluate quantitatively changes in the synergy effects between various muscle groups. For distal shifts of the movement traces, the synergy effects are shown to be changed in different directions, increasing in flexors and decreasing in extensors. The obtained results demonstrate that the muscle hysteresis leads to strong modification of the central commands during movements.

## Introduction

The standard approach for the analysis of central commands in multi-joint movements consists of searching for relationships between kinematic parameters of the movements and electromyograms (EMGs) recorded from the participating muscles. Multiple repetition of the identical movement programs allows for increasing the effectiveness of this analysis by the application of averaging procedures. Examples of this approach can be found in recent studies by our group, in which we considered the circular and linear movement trajectories produced by the right hand in conditions of action of external loads that are directed along the movement traces (Tomiak et al., 2016; Vereshchaka et al., 2018b). In both types of movements, the intensities of EMG activity in the shoulder and elbow muscles are correlated with the respective joint torques, consisting of the positive and negative components, each of which corresponds to the predominant activation of the flexor and extensor muscles, respectively. The methods allowing to evaluate the forces acting on the arm muscles and to define character of the muscle contractions (eccentric vs. concentric) along trajectories of the two-joint movements can be found in earlier studies by our group (Kostyukov, 2016; Lehedza et al., 2016; Lehedza, 2017; Gorkovenko, 2018; Kostyukov and Tomiak, 2018; Vereshchaka et al., 2018a). To distinguish various combinations of activity in muscles acting at different joints, we proposed extracting along the movement paths the segments of coinciding and opposing synergies, with active muscles of different joints belonging to the same (flexor and flexor, extensor and extensor) or opposite (flexor and extensor, extensor and flexor) functionalities, respectively (Tomiak et al., 2015, 2016; Kostyukov and Tomiak, 2018).

Three interdependent types of synergy are usually discussed for description of the human movements: *kinematic*, *kinetic*, and *muscle synergies*. *Kinematic synergies*, representing covariations in the relatively independent changes of the joint angles, are based primarily on anatomical factors in coordinated movements; examples of such synergies are reported, in particular, in studies devoted to manual exploration, and skilled movements (Santello and Soechting, 2000; Thakur et al., 2008). *Kinetic synergies*, which are described by the covariation of forces (or torques) are presented in studies with analysis of the grasping and forced interaction of fingers (Santello and Soechting, 2000; Grinyagin et al., 2005). *Muscle synergies*, which are based on the spatial and temporal coordination of multiple EMGs, have been specifically reported for static hand postures, active force production in the muscles of digits (Weiss and Flanders, 2004; Latash et al., 2007; Castellini and van der Smagt, 2013).

Recent approaches to the study of synergy in real movements, such as locomotion, have use more sophisticated methods, such as principal component analysis (PCA), based on using the linear correlation procedures applied to the EMGs registered in participating muscles (Ivanenko et al., 2004; Wang et al., 2013). At the same time, valid prediction of the central commands and synergy patterns, even in the simplest case of two-joint movements, is not a simple experimental task, especially for complex movement trajectories under actions of changing forces. Recently, we attempted to consider the peculiarities of EMGs in linear parafrontal movements fulfilled under the actions of external loads directed along the movement trajectory (Vereshchaka et al., 2018b). To investigate the observed hysteresis variations of the central commands associated with these movements, we applied a simplified modeling evaluation of the lengths and forces for the muscles participating in the test movements. In the present study, using a similar methodical approach, the experimental procedure was broadened by a comparison of the muscle reactions registered in the parafrontal test movements passing at different distances from the frontal plain. In addition to a natural interest in comparing the linear movements in various zones in the working space, this approach could provide some additional “boundary conditions” that would allow for rechecking the validity of the length and force models for predicting EMG reactions in the muscles under study. Additionally, special attention was paid to analysis of synergies during parafrontal movements and their changes with shifts in traces in the sagittal direction.

### Hypothesis

The central commands to muscles (measured by the surface EMGs) in slow cyclic two-joint movements are closely related to positioning of the following events along the movement trajectory: (1) where the forces generated by the active muscles reverse direction of change; (2) where eccentric contractions are replaced by concentric and vice versa. These events determine EMG hysteresis in cyclic movements; it can be assumed that the hysteresis loops in the flexors and extensors will have opposite directions.

## Materials and Methods

**FIGURE 1:**
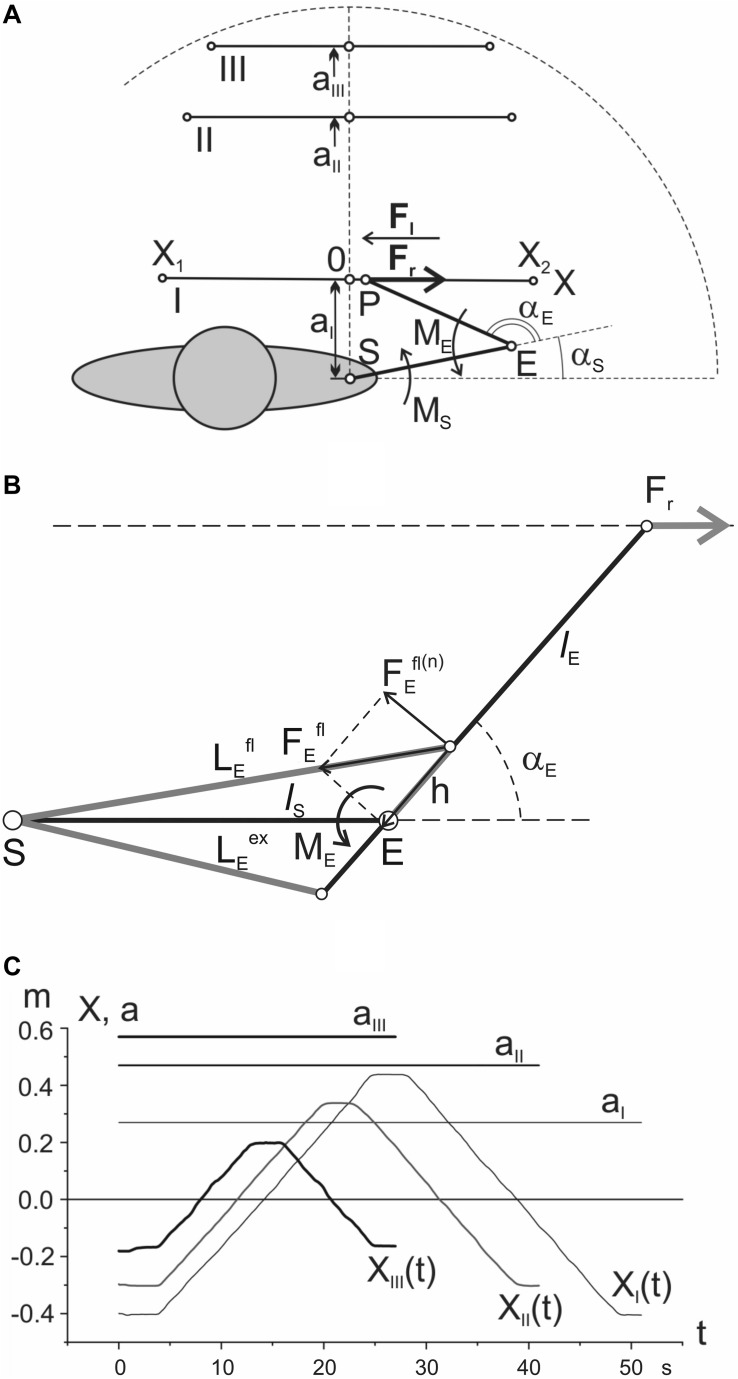
Positioning the movement traces in the working space: **(A)** general scheme of the test movements; **(B)** geometric model describing location of the elbow joint muscles (detailed explanation in the text); **(C)** records of the single movement tests in a real experiment. Notations: I, II, III – proximal, intermediate, and distal traces; X^(1)^ and X^(2)^ – the points restricting test movements; S and E – axes of the shoulder and elbow joints; P – the end point, i.e., the point of force application to the hand; *F*_*r*_ and *F*_*l*_ – the external forces applied to the hand in the rightward/leftward direction, respectively; α_*s*_ and α_*e*_ – the shoulder and elbow joint angles; *M*_*s*_ and *M*_*e*_ – the joint torques around the respective joints. The zero coordinates of the movement traces coincide with respective projections of the shoulder joint axis on these traces. Note that the respective averaged EMGs are also further distinguished by the line thickness in accordance with the used indications of *F*_*r*_ and *F*_*l*_ by thick and thin lines, respectively (see Figure 2). More detailed description is given in the text.

Ten adult men (from 23 to 29 years old), without musculoskeletal or neurological diseases, participated in the experiments. All procedures were fulfilled in framework of the ethical standards of the research committee of Bogomoletz Institute of Physiology, National Academy of Sciences of Ukraine, Kyiv, Ukraine, and with the 1964 Helsinki declaration and its subsequent amendments or comparable ethical standards. Informed written consent was obtained from all participants participating in this investigation. A subject sat before a table on a chair with an adjustable seat height (Figure 1); he gripped by the right hand a handle placed on a moving carriage; the distance between the shoulder joint and the table surface was adjusted in an optimal position for horizontal placement of the entire arm, which was additionally supported at the elbow area by a special belt wrapped around the arm and suspended from a cable to the ceiling of the room. The moving carriage included a system of ball bearings; the movement trajectory was restricted from both sides by parallel metallic rods. Three movement traces were defined by parafrontal lines with approximate distances of 0.27 (I), 0.47 (II), and 0.57 m (III) from the frontal plane passing via both shoulder joints of the subject (Figure 1A). At trace I, the boundaries of movement (X_1_ and X_2_) were placed at distances near 0.4 m on the left and right of point 0, coinciding with a projection of the shoulder joint axis (S) on the movement trajectory. The trajectories X_*I*_(*t*), X_*II*_(*t*), and X_*III*_(*t*) in Figure 1B consisted of slow steady movements from X_1_ to X_2_ points with a velocity of 4 cm/s and stoppage for 3 s at position X_2_, followed by returning to X_1_ with the same velocity as in the first phase of movement. The movement along trace I had a duration near 45 s; test traces II and III were shorter due to an inconvenience of respective movements at the edges, so their durations were approximately 35 and 20 s, respectively. The test movements began under action of the rightward load *F*_*r*_, and they were repeated 10 times with 2-min intervals between tests; then, the load direction changed to the opposite, *F*_*l*_, and the same movements were repeated again 10 times. Subsequently, the same experimental program was applied to movement traces II and III; the rest periods between the group of tests consisted of nearly 5 min.

A potentiometric sensor served for exact positioning of the subject’s hand at the moving carriage (P in Figure 1A); the positional signal was also used for visual tracking of the command showing a desired trajectory of movement on the monitor screen. Two weights (1 kg of mass each) moving vertically were transformed into oppositely directed equal horizontal forces *F*_*r*_ or *F*_*l*_ (10.2 N) by respective systems of cables and pulleys; the forces were applied to the carriage consecutively from the right (*F*_*r*_) and left (*F*_*l*_) sides, thus providing the external forces acting on the hand during the movement tests.

For a qualitative analysis of EMG reactions, it is apparently useful to have at least approximate information on general trends in changes of the muscles’ lengths and forces along various movement traces. Due to the lack of real biomechanical data regarding the geometry of the studied muscles and joints, we can only give a very rough approximation to changes in the corresponding parameters. The simplified geometric diagram in Figure 1B was used to determine the estimated trajectories of length and force in the flexors and extensors of the elbow joint. We described earlier a procedure of computation of the joint torques (*M*_*S*_, *M*_*E*_), and the joint angles (α_*S*_, α_*E*_) for such a model (Vereshchaka et al., 2018b). The length changes for the elbow muscles are defined by the following expressions:

(1) $$L_{E}^{f\,l}=\sqrt{l_{S}^{2}+h^{2}+2\,l_{S}\,h\,{\text{cos }\alpha }_{E}};L_{E}^{e\,x}=\sqrt{l_{S}^{2}+h^{2}-2\,l_{S}\,h\,{\text{cos }\alpha }_{E}},$$

where α_*E*_ is the elbow joint angle; *l*_*S*_ presents the length of the shoulder segment of the arm (i.e., distance SE in Figure 1B); *h* is distance between axis of the elbow joint and point of the flexors and extensors inceptions.

Further, using the proposed earlier methods for definition of the torques $M_{E}^{+};M_{E}^{-}$ (Vereshchaka et al., 2018b), it is possible to define the normal component the forces acting around the elbow joint:

(2) $$F_{E}^{f\,l\,\left(n\right)}=\frac{M_{E}^{+}}{h};F_{E}^{e\,x\,\left(n\right)}=\frac{M_{E}^{-}}{h}$$

Forces $F_{E}^{f\,l},F_{E}^{e\,x}$are calculated using the corresponding trigonometric transformations. The graphs obtained in result of the modeling are shown in Figure 3. Appropriate graphs for the muscles of the shoulder joint were obtained using the obstacle-set method described in detail by Garner and Pandy (2000).

In the present experimental session, two computers were used: one for the visual tracking and another for the data recording. The actual hand movement was displayed in real time by a light spot on the monitor screen; the experimental task consisted of combining this marker with a second one corresponding to a necessary trajectory of movement. The data records included the actual position of the subject’s hand together with EMGs from the eight muscles of the upper limb, namely *m.m. brachioradialis* (*Br*), *biceps brachii caput breve* (*BBcb*), *biceps brachii caput longum* (*BBcl*), *triceps brachii caput laterale* (*TBclat*), *triceps brachii caput longum* (*TBcl*), *ðectoralis major* (*Pm*), *deltoideus pars scapularis* (*Dps*), and *deltoideus pars clavicularis* (*Dpc*). The EMGs were recorded by surface bipolar glued electrodes (Skintact F-301, Austria); an interelectrode distance consisted of 2.0 cm. The bandpass of the amplifiers corresponded to a 0.1–1000 Hz range; the signals were registered with sampling rate 2 × 10^3^ s^–1^ by PCI 6071E and 6023E ADCs (National Instruments, United States). The signal analysis was based on LabView software (National Instruments, United States). The EMG signals were subjected to: (1) high-pass filtering (a fourth-order Butterworth filter with 20 Hz cutoff frequency); (2) full-wave rectification; (3) low-pass filtering (a fourth-order Butterworth filter with 5 Hz cutoff frequency). The EMG signals were normalized with respect to their averaged values recorded during the maximum voluntary contraction (MVC) of the corresponding muscles. All off-line computations were performed using Origin 8.5 (OriginLab Corporation, United States). The averaged EMGs were additionally smoothed using a sliding averaging procedure (window of 200 points). The methods of the computer analysis of the test movements are also described elsewhere (Gorkovenko, 2009, 2018).

Statistical analysis of the EMGs shows that similar characteristics were quite typical for the whole group of subjects (Figure 4A). To compare the EMG intensities in various muscles, the average levels of EMGs were defined using a standard procedure of integration:

(3) $${\bar{E}}_{i}=\frac{1}{T}\,\int_{0}^{T}E_{i}\,\left(t\right)\,d\,t,$$

where *E*_*i*_(*t*) is the current EMG intensity in the *i-*th muscle, and *T* is the test duration.

Quantitative analysis is related to the hysteresis evaluation of the EMGs in the test movements. The following expression, proposed in our preceding study (Vereshchaka et al., 2018a), was used for computation of the normalized areas of the EMG loops:

(4) $$H_{i}^{\left(n\right)}=\frac{\int_{X_{1}}^{X_{2}}E_{i}\,\left(X\right)\,d\,X-\int_{X_{2}}^{X_{1}}E_{i}\,\left(X\right)\,d\,X}{\left(X_{2}-X_{1}\right)\,{\bar{E}}_{i}},$$

where the integrals define the areas under EMG recording in the direct and reversed-phases of the test movements; ${\bar{E}}_{i}$is defined by Eq. 3.

The statistical analysis was performed by ANOVA with repeated measurements. Two within-group factors were considered: (i) distance; and (ii) direction of the applied force. The first factor had three levels, namely, proximal (I), medial (II), and distal (III), whereas the second had only two – the force directions *F*_*r*_ and *F*_*l*_. *Post hoc* analysis was performed using Bonferroni’s test. Intergroup differences were supposed significant at *P* ≤ 0.05; software SPSS Statistics (17.0 version, IBM Analytics, United States) was used for statistical calculations.

## Results

**FIGURE 2:**
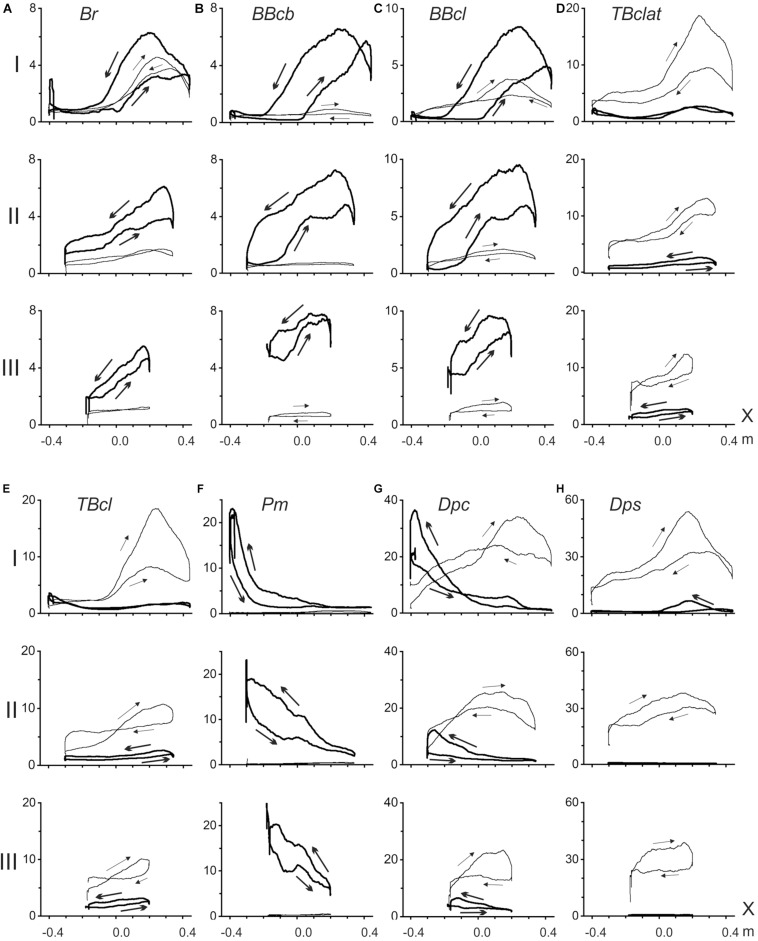
Averaged EMGs recorded from the elbow **(A–E)** and shoulder **(F–H)** muscles during ten repetitions of the standard test movements with right- and leftward external loads (*F*_*r*_ and *F*_*l*_), as shown in Figure 1; the records corresponding to *F*_*r*_ and *F*_*l*_ forces are shown by thick and thin lines, respectively. Abscissa in all plots (X) define positioning along the movement trace. Arrows on the recorded EMG loops signify their circumvention directions in a standard sequence of the alternating test movements X^(1)^→X^(2)^→X^(1)^. EMG intensities are presented in% of the MVC, defined for each of the muscles during their maximal isometric contractions before the main test procedure.

The present methodical approach allows to analyze: (1) the EMGs recorded from the muscles of the elbow and shoulder joints during slow parafrontal alternating movements (the position – EMG intensity hysteresis); (2) dependence of EMG patterns on the load direction; and (3) changes in the EMG patterns with increased distance of the movement traces in the sagittal direction (Figure 2). The hysteresis type of EMG activity is well seen in both the shoulder and elbow muscles; at the same time, the shape and amplitude parameters of the respective hysteresis loops markedly change during sagittal shifts of the movement traces: I→II→III. In movement tests with application of the rightward forces (*F*_*r*_), the flexor muscles, both in the elbow and the shoulder, showed predominant activation of the flexors, while extensors were either entirely passive or showed weak coactivation with lesser EMG amplitudes. In contrast, in the presence of leftward forces (*F*_*l*_), extensors were predominantly activated, whereas flexors remained inactive. In the subject, these reactions are presented in Figure 2; a deviation from the above simplified scheme might be noted in the activities of *Br* and *BBcl* for the test movements passing most closely to the trunk (trace I). In this case, rather strong cocontraction activity of these muscles is observed under action of the *F*_*l*_ forces. However, the cocontraction almost disappears in transitions to distal traces II and III. A similar well-expressed deviation could be found in reactions of the shoulder muscle *Dpc* (Figure 2G); moreover, in distal traces II and III, reactions of this muscle during the action of *F*_*l*_ forces even exceeded those recorded under application of *F*_*r*_ loads. Most likely, such a “mixed” type of reaction in the *Dpc* muscle can be associated with the presence of its individual parts involved in flexion and extension movements.

Based on the positional arrangement of the EMG focusing on the muscles of different functional groups, some overlapping could be noted of the activation zones in both the flexors and extensors of the elbow joint, as well as in the shoulder extensors. As clearly seen on the proximal movements (I), a more noticeable activity in these muscles is associated with the right halves of the tracks (Figures 2A–E,H), while the shoulder flexors show higher levels of activation at the left parts of the tracks (Figure 2F). A similar “left-right” separation between focuses of activity during the action of *F*_*r*_ and *F*_*l*_ loads is also well observed in reactions of the “mixed” shoulder muscle *Dpc* (Figure 2G).

All of the recorded EMGs demonstrate well-expressed hysteresis. For *F*_*r*_ and *F*_*l*_ loads evoking predominant reactions in the flexors and extensors, the correspondent hysteresis loops are directed oppositely to each other. In flexors (*F*_*r*_ loads) and extensors (*F*_*l*_ loads), the loops always have anticlockwise and clockwise directions, respectively (Figure 2).

**FIGURE 3:**
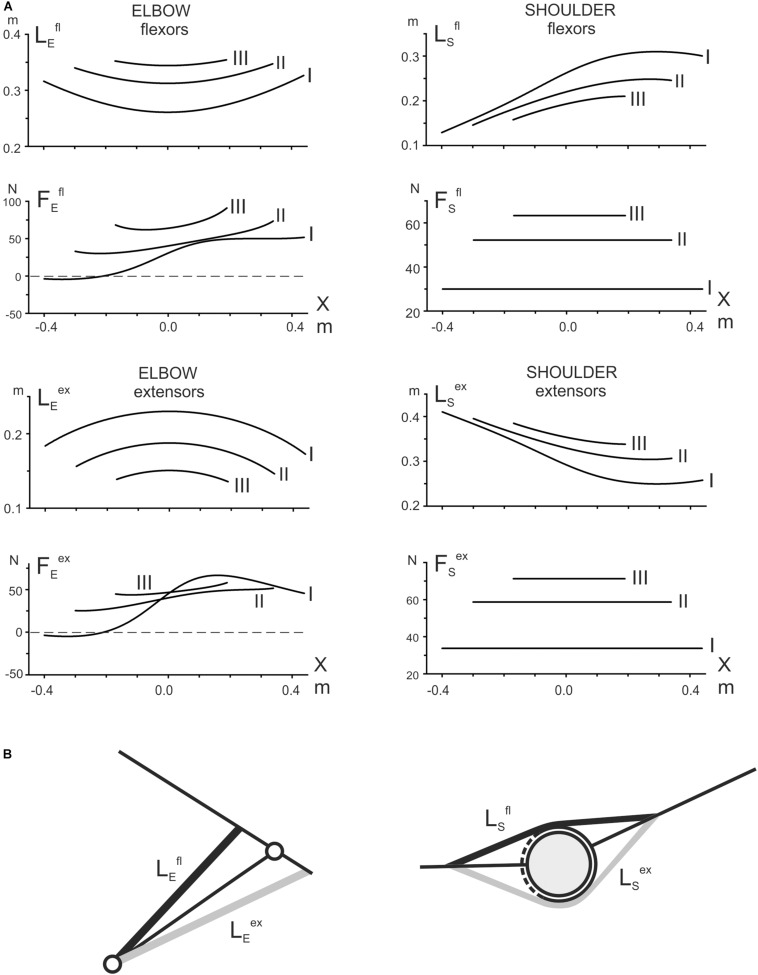
**(A)** Theoretical evaluation of the length changes in the elbow and shoulder muscles and prediction of the forces acting on them in the test movements. For simplicity, only the single-joint muscles are modeled **(B)**; a straight-line model (left panel in **B**) has been applied to simulate the elbow muscles; the shoulder muscles are modeled using the obstacle-set method (Garner and Pandy, 2000) (right panel in **B**). It should be noted that the simulated curves are inevitably very approximate due to the lack of basic anatomical parameters. Nonetheless, it is assumed that the obtained plots could provide a general impression about the character of the parameter changes during the test movements, thus helping to treat qualitatively the registered EMG traces (Figure 2).

For a simplified simulation of the length and strength of the muscles involved in test movements, we limited the consideration to the muscles that work in each of the two joints in isolation (Figure 3). At least for the elbow joint, such a restriction seems to be quite reasonable due to the high extent of similarity in reactions of the two- and single-joint muscles in both flexors and extensors (compare the pairs *BBcb* and *BBcl* and *TBclat* and *TBcl* in Figure 2). To simulate the muscles in the elbow joint, its straight-line scheme (Figure 1B) was served; for the shoulder muscles, we applied the obstacle-set method described in detail by Garner and Pandy (Garner and Pandy, 2000; Figure 3B). The simulation results shown are inevitably approximate; however, the plots can create a general impression about the tendencies for changing the parameters within the three tests I–III. At least for the muscle lengths, one can compare the plots with a visual inspection of their possible changes at various end-point positions of the movement traces.

The simulation presented in Figure 3A demonstrates significant differences in the changes of the corresponding parameters belonging to the muscles of two joints. First, considering the length changes with respect to positioning along the movement tracks, we can point out their symmetry with the elbow muscles and asymmetry with the shoulder muscles. Second, comparing the length changes in transition to more distal traces, one can note the opposite direction of their changes for identical functional groups of the muscles at different joints; the elbow flexors are elongating, whereas the shoulder flexors are shortening; the extensors demonstrate the reverse order of change. Third, the parafrontal movement traces are connected to the constancy of the forces acting on the shoulder muscles, both flexors and extensors, while the forces acting on the elbow muscles are essentially non-linear; at the same time, the forces acting on the muscles of both joints have similar tendencies to an increase in the distal shift of the movement traces.

All of the averaged EMG records registered in the muscles of both joints demonstrate evident hysteresis in the alternating test movements (Figure 2). The position – EMG intensity loops, which are registered in the muscles during the action of the forces evoking their maximal activation (*F*_*r*_ in flexors and *F*_*l*_ in extensors), have directions that are strictly dependent on their functional belonging: counterclockwise in flexors and clockwise in extensors. Therefore, the direction of the EMG hysteresis loops is dependent on the force direction. At the same time, the real shapes of the hysteresis loops are assumed to be closely connected to presumable changes in the muscle lengths and the forces acting on the muscles, which are evaluated by the above simulation (Figure 3). First, we can consider the reactions of the shoulder muscles, in which the acting forces are presented by the constant lines shifting vertically for more distal traces (Figure 3). Hence, the shoulder muscles, both flexors and extensors, are contracting in these cases in isotonic conditions. All of the test movements (I–III) begin with opposing of the shoulder flexors to the external load; these muscles should develop some initial efforts, thus generating noticeable EMG activities (Figure 2F). Further movements in tests I–III are accompanied by isotonic lengthening of these muscles; because the lengthening muscles create greater forces, the EMG intensities in these muscles quickly decrease. Moreover, due to a decrease in the lengthening velocity in consecutive tests I–III (*L_*s*_^*fl*^* in Figure 3A), we can observe a rate lowering of the EMG decrease in traces II and III at the beginning of the movement tests (Figure 2F). In contrast, the reversed-phases of movement proceed by shortening these muscles, demanding an additional inflow of excitation; therefore, the returning EMG branches go above the direct ones. Therefore, in coordinates, the end-point position – the EMG intensity – the hysteresis loops have a counterclockwise direction.

The shoulder extensors change their lengths oppositely to the flexors (*L_*s*_^*ex*^* in Figure 3A). These muscles are shortened during the direct movement phase and lengthened in the reverse phrase, leading to a change in the hysteresis direction compared with the flexors (Figure 2H). The “mixed” muscle *Dpc* (Figure 2G) demonstrates the presence of two hysteresis loops, each of which corresponds to an action of the relating force direction. The directions of the loops are defined by the functionality of correspondent muscle compartments; i.e., they are counterclockwise for the flexor parts and clockwise for the extensor parts.

It can be expected that the elbow muscles can exhibit somewhat more complex EMG reactions, likely because the forces acting on these muscles also change together with the changes in their lengths. Therefore, instead of isotonic contractions as in the shoulder muscles, the external forces acting on the elbow muscles are also changing. Moreover, the positional dependencies of the forces also demonstrate complex modifications with distal shifts of the traces. The loading curves, which are reconstructed by the simulation for both the elbow flexors and extensors, point out on the presence of negativity at the beginning of the most proximal test movement (I), which could signify the absence of loading for these muscles at the beginning of movement, in turn explaining the retardation of the EMG reactions in these muscles (Figures 2A–E). In elbow flexors, a rise of activity in the middle part of the trace can be accompanied by lengthening of the muscles. A stoppage at the right end of the trace decreases the EMG intensities in the elbow flexors, whereas the following reverse movement, accompanied by active muscle shortening, evokes a rapid rise in the EMGs. Thus, the complex interaction of changes in load and length is likely the main reason for the appearance of a local maximum in the EMG activity record, followed by its consequent lowering. The hysteresis loops in the both biceps heads show a clear tendency for increasing in height in more distal movement traces (II, III), whereas reactions of the brachioradialis do not show similar growth. This finding might be connected to an evident discrepancy of this muscle with the biceps model, predicting heightening in both the muscle length traces and respective loading curves in distal movement tests II and III (Figure 3).

The elbow extensors, in general, are predominantly subjected to the action of the *F*_*l*_ forces; under the condition of the alternating test movements, both triceps heads demonstrate quite similar hysteresis loops having opposite direction in respect to the flexors loops recorded under the action of *F*_*r*_ forces. In contrast, in transition to more distal traces, the EMG reactions are decreasing, and respective hysteresis loops are decreasing, which is essentially different from the EMG activities in flexors. However, it could be easily explained whether these reactions would be analyzed using the respective modeled length and load traces (Figure 3A). In extensors, the length traces (*L_*E*_^*ex*^*) decrease in more distal traces; i.e., this order is the opposite of that in the flexors. Due to the proximity of the respective force dependencies (*F_*E*_^*ex*^*), the character of the length changes could play a key role in the above described evolution of the EMG hysteresis loops.

It seems to be important that, in the cases of a well-expressed cocontraction of the antagonists during the action of forces evoking predominant activity on the agonists, the directions of the smaller hysteresis loops in the antagonists are always opposite to the directions of the larger loops registered in the predominant type reactions of the agonists. This important property of hysteresis in the antagonistic muscle groups manifests itself in both the elbow and shoulder muscles, and it is applicable to both flexors and extensors.

**FIGURE 4:**
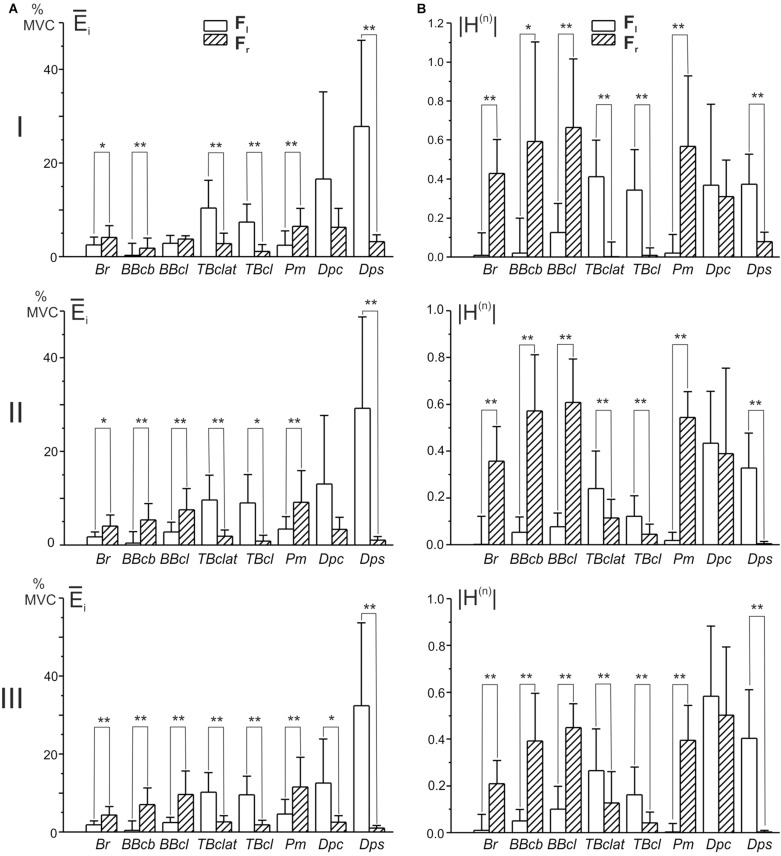
Diagrams of the mean EMG magnitudes **(A)** and the normalized areas of the position – EMG intensity hysteresis loops **(B)** defined in the group of ten subjects. EMGs were registered in each subject during movements fulfilled under consecutive action of the oppositely directed loads *F*_*r*_ and *F*_*l*_ (ten repetitions of each test); the parameters were calculated using Eqs 3 and 4, respectively. Due to a difference in the signs of the normalized areas of the hysteresis loops, their absolute values |*H*^(^*^*n*^*^)^| were used for statistical comparisons. Asterisks designate cases in which the difference in correspondent parameters was statistically significant for different loads (*F*_*r*_ and *F*_*l*_) (paired *t*-test, ^∗^*P* < 0.05, ^∗∗^*P* < 0.01).

In accordance with the graphs presented in Figure 4A, EMG reactions are better expressed in the flexors of both joints, when external force is directed to the right (*F*_*r*_, dash bars); the extensors demonstrate a more noticeable activity for opposite loads (*F*_*l*_, open bars). The statistically significant difference between EMGs at different loads was not observed only in the shoulder muscle *Dpc* (tests I and II) but also in the elbow flexor *BBcl* (test I). In the first case, such a reaction of the *Dpc* muscle, as we had already pointed out above (see description of Figure 2), might likely be connected with the complex composition of this muscle, containing both flexor and extensor components. The absence of a difference in the *BBcl* muscle was observed only in test I and could be related to local variability in the activation of this muscle in different subjects.

One can see that *H*_*i*_^(^*^*n*^*^)^ is equal to zero when the direct and return segments of the EMG traces coincide with each other, whereas this parameter obtains negative (positive) values for the counterclockwise (clockwise) hysteresis loops. Due to a difference in the signs of *H*_*i*_^(^*^*n*^*^)^ for the oppositely directed loads, their absolute values |*H*_*i*_^(^*^*n*^*^)^| are used for statistical comparison of the hysteresis effects in these cases (Figure 4B). In all of the studied muscles, excluding the “mixed” *Dpc*, it is possible to observe an exceeding of this parameter in the reactions of muscles that are the primary “realizers” in a given movement task, fulfilled under the action of a predominant force, *F*_*r*_ in flexors and *F*_*l*_ in extensors. Therefore, the hysteresis effects are better expressed in the primer “realizers” and are smaller in the cocontracting “assistants,” and these differences are strictly associated with the respective hysteresis reversals (Figure 4B).

The results of statistical analysis of the averaged EMGs and of the normalized areas of the hysteresis loops by ANOVA with repeated measurements are summated in Tables 1, 2. Two within-group factors are considered: (1) the distance of the movement traces from the frontal plane; and (2) the direction of the applied force; additionally, a possibility for the interaction of the factors is analyzed as well. The first factor includes three levels of change depending on their positions in the working space (I–III); the second factor consists of two levels depending on the force directions (*F*_*r*_, *F*_*l*_). The analysis demonstrates the strongest action of the force direction factor on both the averaged EMGs and their hysteresis properties (Tables 1, 2); in both cases, statistically significant effects are shown for the respective actions on seven of the eight muscles. Uncertainties relating to the *Dpc* muscle are likely provided by the mixed composition of this muscle, as pointed out earlier. The distance factor exerts weaker influences on the analyzed parameters of four and three muscles, respectively, in Tables 1, 2. A weakness of these effects could constitute the key reason for the relative ambiguity in the interaction of the factors, when statistical significance is registered in only half of the muscles for the averaged EMGs (Table 1) and in a single case for the EMG hysteresis (Table 2).

**TABLE 1:** Statistical analysis of the averaged EMGs (defined by Eq. 3) by ANOVA with repeated measurements for the group of ten subjects.

**Muscles**	**DIST**			**DIR**			**DIST × DIR**		
	**df**	***F***	***p***	**df**	***F***	***p***	**df**	***F***	***p***
Br	2	1.546	0.247	1	11.495	**0.012**	2	6.095	**0.012**
BBcb	2	21.531	**0.000**	1	40.993	**0.000**	2	16.764	**0.000**
BBcl	2	6.719	**0.009**	1	19.796	**0.003**	2	8.976	**0.003**
TBclat	2	3.391	0.063	1	24.965	**0.002**	2	0.021	0.979
TBcl	2	3.438	0.061	1	21.460	**0.002**	2	1.100	0.360
Pm	2	6.328	**0.011**	1	17.123	**0.004**	2	2.933	0.086
Dpc	2	6.606	**0.010**	1	4.980	0.061	2	0.046	0.955
Dps	2	3.534	0.057	1	16.547	**0.005**	2	13.201	**0.001**

*Three within-group factors are considered: the distance of the movement traces from the frontal plane (DIST), the direction of the applied force (DIR), and the interaction of the factors: DIST × DIR. The first factor includes three levels of change depending on the position of the traces in the working space: proximal (I), medial (II), and distal (III); the second factor consists of two levels: right- and leftward directions of the force (*F*_*r*_, *F*_*l*_). *Post hoc* analysis is based on the Bonferroni’s test; the intergroup differences are supposed to be statistically significant at *p* ≤ 0.05 (the cells marked by bold fonts).*

**TABLE 2:** Statistical analysis of the normalized areas of the hysteresis loops (defined by Eq. 4) by ANOVA with repeated measurements for the group of ten subjects.

**Muscles**	**DIST**			**DIR**			**DIST × DIR**		
	**df**	***F***	***p***	**df**	***F***	***p***	**df**	***F***	***p***
Br	2	10.051	**0.002**	1	17.528	**0.004**	2	4.523	**0.031**
BBcb	2	2.871	0.090	1	5.591	**0.050**	2	3.419	0.062
BBcl	2	1.721	0.215	1	49.860	**0.000**	2	0.209	0.814
TBclat	2	14.169	**0.000**	1	31.386	**0.001**	2	1.292	0.306
TBcl	2	6.879	**0.008**	1	24.166	**0.002**	2	3.209	0.071
Pm	2	2.856	0.091	1	55.503	**0.000**	2	0.728	0.500
Dpc	2	0.183	0.834	1	13.108	0.09	2	1.208	0.328
Dps	2	1.570	0.243	1	35.489	**0.001**	2	3.720	0.051

*Three within-group factors are considered: the distance of the movement traces from the frontal plane (DIST), the direction of the applied force (DIR), and the interaction of the factors: DIST × DIR. The first factor includes three levels of change depending on the position of the traces in the working space: proximal (I), medial (II), and distal (III); the second factor consists of two levels: right- and leftward directions of the force (*F*_*r*_, *F*_*l*_). *Post hoc* analysis is based on the Bonferroni’s test; the intergroup differences are supposed to be statistically significant at *p* ≤ 0.05 (the cells marked by bold fonts).*

**FIGURE 5:**
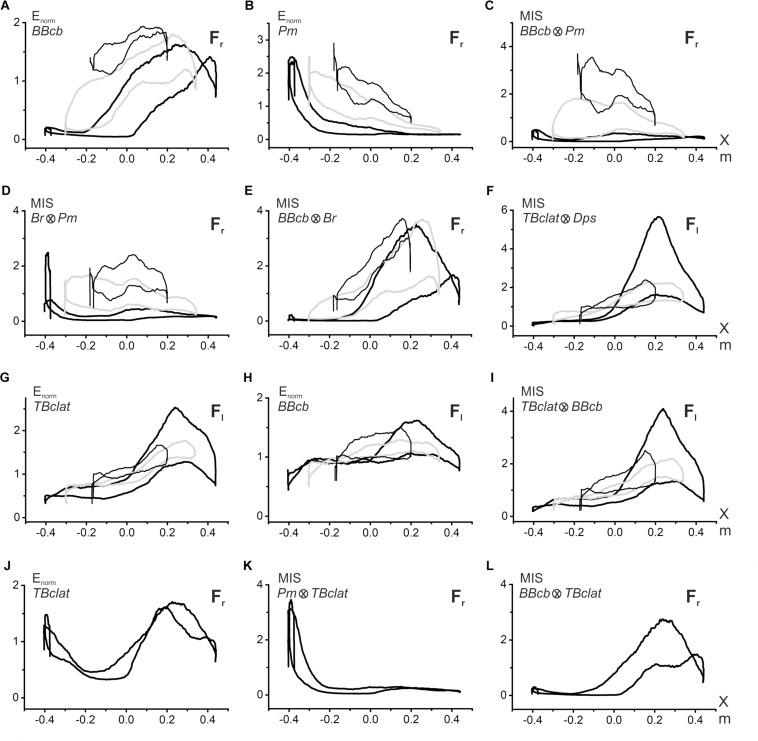
Evaluation of the synergic interaction of the muscles belonging to the same or different joints during fulfillment of the parafrontal test movements. Procedure for computation of the multiplication index of synergy (MIS) is explained in the text. The graphs related to different movement tests I–III are distinguished in plots **(A–I)** by the following lines: I – thick, black; II – thick, gray; III – thin, black; the plots **(J–L)** contain only test I. Symbols F_*r*_, F_*l*_ in the right corner of each plot signify direction (right- or leftward) of the external force acting on the subject’s hand. The data were obtained from the same subject as in Figure 2.

The characteristics of the EMG activities presented above demonstrate the predominance of the coinciding pattern of synergy in the activity of the muscles belonging to different joints. When the external force has a rightward direction, the flexor muscles are more active; oppositely, the leftward forces evoke a prevailing activation of the extensors. However, such a description is likely oversimplified, and it does not consider possible position-dependent variances in the relationship between the EMG intensities of the muscles belonging to different joints; moreover, the respective EMG loops are deformed in different ways in transition among the movement traces (I–III). Below we propose a possible approach to evaluate quantitatively the synergy interaction between two different muscles (Figure 5). Its essence is demonstrated in Figures 5A–C. Panels A and B present superpositions of the normalized EMG activities of the elbow and shoulder flexors (*BBcb* and *Pm*) registered in movement tests I–III (the data from the experiment are shown in Figure 2). The normalization of the corresponding EMG loops is produced regarding the following mean values.

(5) $${\bar{E}}_{i}^{\left(\text{Σ}\right)}=\frac{1}{3}\,\sum_{j}{\bar{E}}_{i}^{\left(j\right)},$$

where ${\bar{E}}_{i}^{\left(j\right)}\left(j=I,\mathrm{II},\mathrm{III}\right)$ are defined by Eq. 3 for *i*-th muscle in each of the movement tests (I–III).

The extent of the synergic interaction of the muscles can be quantified by multiplication of the respective normalized averaged EMG records in the *i-* and *k-*th muscles, which will be called hereafter the multiplication index of synergy or MIS:

(6) $${\text{MIS}}_{i,k}\,\left(t\right)=E_{i}⊗E_{k}=\frac{E_{i}\,\left(t\right)\,\text{⋅}\,E_{k}\,\left(t\right)}{{\bar{E}}_{i}^{\left(\text{∑}\right)}\,\text{⋅}\,{\bar{E}}_{k}^{\left(\text{∑}\right)}}$$

Under the conditions of the steady linear movement, MIS(*t*) can be presented in the form of the coordinate dependence, MIS(X) (Figure 5C). As with the original *E*(X) records, the MIS(X) traces have the appearance of hysteresis loops, showing a striking difference for successive movement tests. These loops have minimal sizes in most proximal movements (I); their areas increase in more distal tracks II and III, shifting upward in the latter case. It seems that the observed pattern of the MIS(X) loops satisfactorily reflects peculiarities of the synergic interaction between the elbow and shoulder flexors. In most proximal movements, the activities in the shoulder and elbow flexors are predominantly distributed over different parts of the trace; if *BBcb* is activated mostly in the middle and right parts of the trace, the *Pm* activity is located predominantly on the left side and drops down on the right. Such a separation of activity zones leads to an insignificant expression of synergy effects. In contrast, distal shifts to tracks II and III cause a noticeable overlapping of the zones therefore, expression of the synergy effects becomes more visible (Figure 5C). The coincidence of the hysteresis directions in the flexor muscles of both joints leads to forming the same counterclockwise direction of the MIS(X) loops describing synergic interaction of these muscles. The opposite slopes of the hysteresis loops in *BBcb* and *Pm* muscles can diminish their expression in the MIS(X) loops, which are oriented mostly horizontally. A similar pattern of the MIS(X) loops is also observed in other combinations of the agonist muscles, for example, *Br* and *Pm* (Figure 5D). Quite strong synergy effects can be observed when comparing activity in close agonists belonging to the same joint, as in the case of *BBcb* and *Br* (Figure 5E). It can also be indicated that the synergies are manifested differently in the extensors belonging to the elbow and shoulder (Figure 5F), compared to the flexor pairs described above (Figures 5C,D). In the combination of the *TBclat* and *Dps* muscles, the strongest synergy effects are observed for proximal movements (I), and these effects are similarly reduced in both distal traces II and III (Figure 5F). This character of synergy is likely related to the close similarity of the EMG reactions in the extensor muscles belonging to different joints (Figures 2D,H).

The proposed approach can also be applied to assess synergies between the predominant activation of the extensor muscles and the weak cocontractions of the elbow flexors that accompany this activity (Figures 5G–I). The *MIS* loops registered in this case (Figure 5I) demonstrate a general resemblance with the origin EMG loops in the extensors (Figure 5G); the directions of the hysteresis loops coincide with each other. In some cases, the proposed method allows for separating different synergic components in the cocontractions of the antagonist muscle, for example, *TBclat* in Figure 5J. The first component might be treated as a response to the activity of the agonist belonging to a given joint (*BBcb*, Figure 5L), while the second one presents the reaction to the contraction of the agonist muscle acting at another joint (*Pm*, Figure 5K).

## Discussion

In the present work, earlier developed methods for the analysis of parafrontal hand movements (Vereshchaka et al., 2018a) have been extended to compare traces with different distances from the subject’s body. This extension allowed for applying multifactor ANOVA to describe quantitatively the EMG activities in the muscles providing these movements. Additionally, our analysis was enlarged by simulation of the positional dependencies of the muscle lengths and acting forces; despite the inevitably approximate character of the parameter estimation, comparison of three different movement traces allowed for increasing the effectiveness of such a simulation for the treatment of the positional and load-dependent changes in the registered EMGs. The general features of the corresponding EMG reactions were considered from the point of view of the non-linear muscular dynamics, including hysteresis-like muscular behavior (Kostyukov, 1998).

We can see that the muscles of the coinciding functional modality (flexors, extensors) belonging to different joints, demonstrate a definite similarity of their hysteresis properties. Flexors of both joints react predominantly on the *F*_*r*_ loads; reactions of extensors are maximal during the action of the *F*_*l*_ loads, and the respective hysteresis loops registered under the action of these forces are counterclockwise (clockwise) in the flexors (extensors). The agonist muscles for given loads (*F*_*r*_ for flexors and for *F*_*l*_ for extensors) are activated in the cocontraction modes during action of the opposite loads; it seems to be important that the hysteresis loops reverse their direction compared with the predominant patterns of activation. Statistical analysis of the EMG amplitudes and related hysteresis effects by ANOVA with repeated measurement demonstrates the strongest action of the force direction factor in both cases; the distance factor exerts a weaker influence on the parameters (Tables 1, 2).

Earlier, we suggested that the synergy of activation, reflecting the simultaneous activation of muscles belonging to different joints, might be closely related to the patterns of coincidence/opposition in the directions of the torques around the corresponding joints (Kostyukov, 2016; Tomiak et al., 2016; Gorkovenko, 2018; Kostyukov and Tomiak, 2018). Therefore, the existence might be proposed of a close relationship between the activation and force synergies. At the same time, the present results show a presence of rather noticeable changes in the EMG intensities in both flexors and extensors of the shoulder joint (Figures 2F,H), whereas the shoulder torques and appropriate forces, defined by the simulation procedure, remain unvaried. Hypothetically, such a result might be explained by strong length-dependent changes in the contraction forces of the shoulder muscles.

Despite essential positional influences on the EMGs in the muscles providing two-joint movements, the spatial distributions of the forces acting on different muscles and their synergic characteristics could play a predominant role in forming the synergies of the related central processes. In our previous theoretical study (Kostyukov and Tomiak, 2018), we classified two types of the force synergy in accordance with simultaneous excitation of the muscles belonging to different joints. The *coinciding synergy* relates to the similar modalities of the activated muscles at the joints (flexors and flexors; extensors and extensors), the *opposing synergy* matches combination of the different modalities (flexors and extensors; extensors and flexors). It has been shown theoretically that there is a strict prevalence of the coinciding synergy for isometric two-joint muscle contractions with complete circle turning of the end-point force vector within the working space (see Figure 6 in Kostyukov and Tomiak, 2018). The present study demonstrates experimentally the strong predominance of coincident synergy in real parafrontal movements produced in different parts of the working space. In fact, this type of linear movement is realized through the activation of flexors or extensors belonging to both joints, while a change in the direction of externally applied forces causes an exchange between the same combinations of muscles. It can be assumed that such a predominance of coincident synergistic patterns is simply associated with purely geometric constraints for the joint movements. It seems likely that equivalence between the two types of synergy can be achieved only in the purely hypothetical case of complete, unrestricted rotation in the joints, which is impossible in reality.

The present study demonstrated the existence of differences in the directions of the position – EMG hysteresis loops in the flexor and extensor muscles of both joints under the action of right- or leftward loads. This difference is easily explained by the general characteristics of muscle hysteresis (Kostyukov, 1998) in combination with the opposite directions of the length changes in the muscles – antagonists (Figure 3A). At the same time, it seems to be greatly important for the comparison of similar movement trajectories in different places within the working space; one can be assured that the central programs in the flexor and extensors will change in quite different ways, and this concern of muscles belongs to different joints (Figure 2). At least the transformation of the EMG hysteresis loops in antagonists for such trajectories can be easily explained by the simulation plots of the muscle length changes (Figure 3A). During distal shifts of the movement trajectories, despite a general conservation of the shapes of length traces, they shift in opposite direction in flexors and extensors. Whereas the length traces in the elbow (shoulder) flexors move in the direction of the muscles that are lengthening (shortening), the correspondent traces in the extensors shift in the opposite direction.

A complex transformation of EMG loops in the muscles of both joints indicates changes in their synergistic interaction. To describe quantitatively the extent of synergic interaction between muscles, we proposed using the MIS approach. During shifts of the movement trajectories in the sagittal direction, we noted essential differences in the MIS transformations (Figures 5C,F). In the flexor pairs, the MIS loops change at very low levels in the proximal movements; their ranges are essentially raised in the middle and distal trajectories. In contrast, in extensors, the MIS loops reach maximum values ιn proximal movements, decreasing in more distal traces. The directions of the MIS loops coincide with the directions of the respective EMG loops, showing a similar reversal for the cocontraction modes of activity in the muscle under study.

The interaction of agonistic muscles at both joints can create a powerful source of uncertainty in two-joint movements. Opposite length changes in antagonistic muscles during movements should essentially modify the hysteresis after-effects (Kostyukov, 1987, 1998; Kostyukov and Korchak, 1997; Gorkovenko et al., 2012). In studying the postural movements, it has been shown that peoples often use muscle cocontraction to stabilize the limb joints in the presence of load disturbances (Milner and Cloutier, 1998). In most cases, people can independently control the relative balance of cocontraction in antagonist muscles, thus varying the stiffness of joints over broad limits (Gribble and Ostry, 1998; Burde et al., 2001; Kostyukov, 2016). In our study, we observed a direction change in EMG hysteresis when the muscles from the active contraction program assisted their antagonists in cocontraction mode. At the same time, cocontraction of the antagonists will increase energy costs for real movements (Kostyukov, 1998; Burde et al., 2001).

A simplified modeling of the muscle lengths and forces in various test movements seems to be very useful for qualitative analysis of the averaged EMGs. At the same time, we understand that such a modeling is not sufficiently precise and can present only rough evaluations of the parameters. The real biomechanics of the elbow and shoulder joints could introduce a significant inaccuracy in the calculation of both the acting forces and the muscle lengths. The rotation geometry in the shoulder joint is known to be extremely complex (Hill et al., 2008); movements around the elbow joint, which may be presented by a bunch of three interactive elements (Bryce and Armstrong, 2008), are not simpler as well. Our simplified model also does not consider participation of the biarticular muscles; it is much more difficult to define the places of force application in this case (Pigeon et al., 1996; Van Bolhuis et al., 1998). Additionally, our experimental model corresponds only to slow movements with constant velocity; therefore, any real movement with changing velocity will inevitably need dynamic methods of analysis (Hollerbach, 1982; Wolpert and Kawato, 1998; Kawato, 1999; Wolpert and Ghahramani, 2000).

The present results seem to be consistent with the “leading joint” hypothesis proposed by Dounskaia (2005) for the analysis of the multi-joint movements. This hypothesis suggests that a complex multi-joint movement can be somewhat simplified by choosing a “leading joint,” then analyzing the entire movement on this basis. On the upper limb, the shoulder joint is most suited for this role because of the greater inertia of the proximal part in the upper limb (Dounskaia and Wang, 2014).

Currently, the term “synergy” allows for multivalued treatment, which depends on the complexity level of the considered motor tasks; exhaustive consideration of the problems can be found in a number of theoretical studies (Latash, 2010, 2012; Bruton and O’Dwyer, 2018). It is likely that the simplest type of synergy can belong to the single-joint movements, in which it is possible to consider the synergic interactions between agonists and to evaluate the role of cocontractions of the antagonists (Gorkovenko et al., 2012). The two-joint movements (Tomiak et al., 2015, 2016; Kostyukov and Tomiak, 2018) constitute the next level of synergic interaction of the muscles during fulfillment of more complex movement tasks with arbitrary planar transitions of the end-points produced under the loading action of externally applied forces. The synergy effects in the above simplest types of arbitrary movements cannot be treated in terms of reduction in the number of degrees of freedom (Tresch and Jarc, 2009). Similarly, the constituent variables related to the single- or two-joint movements are hardly to be considered task-specific covariations stabilizing the output performance of the motor control system (Latash, 2010). Conversely, the present results demonstrate the presence of fundamental non-linear components in the synergic interactions between muscles. The synergy analysis in real movements, such as locomotion, use the PCA method based on the correlation procedures applied to the EMGs in the muscles participating in a given movement program (Ivanenko et al., 2004; Wang et al., 2013). Evident shortcoming for this approach may consist in application of the linear correlation methods for analysis of a fundamentally non-linear system. EMG hysteresis is directly related to muscle hysteresis *per se*, which reflects the non-linear relationships of the activation intensity coming to the muscle with its length and force (Kostyukov, 1998). Consequently, the EMG hysteresis inevitably contains static, non-linear components that must cover the low frequency range of motor commands in the frequency domain, thus distorting the synergy analysis and phase shifts grounded in the decomposition algorithms (Bruton and O’Dwyer, 2018).

## Conclusion

The averaged EMGs were registered from the arm muscles in isotonic parafrontal movements of the right hand in the horizontal plane. Slow alternating hand transitions, directed first on the right and then on the left, were produced under action of the isotonic loads applied to the hand in the right- and leftward directions. The elbow and shoulder flexors reacted predominantly on the rightward loads; the extensors were mostly activated by the leftward loads. The averaged EMGs in both flexor and extensor muscles belonging to different joints demonstrated hysteresis properties; the counterclockwise direction of the hysteresis loops was always registered in flexors and the clockwise direction in extensors. The muscles predominantly opposing the loading forces of a given direction participate in a cocontraction mode as antagonists when the direction of load was changed; together with a decrease in the amplitude of the hysteresis loops, their direction was also reversed. The MIS has been proposed to evaluate quantitatively changes in the synergy effects between various muscle groups. For distal shifts of the parafrontal movement traces, the synergy effects are shown to be changed in different directions, increasing in flexors and decreasing in extensors. The obtained results demonstrate that the muscle hysteresis lead to strong modification of the central commands during movements.

## Data Availability Statement

The datasets generated for this study are available on request to the corresponding author.

## Ethics Statement

The studies involving human participants were reviewed and approved by the Ethics Committee of A.A. Bogomoletz Institute of Physiology, National Academy of Sciences of Ukraine, Kyiv, Ukraine. The participants provided their written informed consent to participate in this study.

## Author Contributions

AK contributed to the idea of the study and the writing. OL and TA contributed to the participation in the experiments and the data handling. AG contributed to the computer procuring and statistical treatment of results. WP contributed to the participation in experiments and the discussion. VM contributed to the discussion of the results. MZ contributed to the discussion of the results and organization of the financial support for the project.

## Conflict of Interest

The authors declare that the research was conducted in the absence of any commercial or financial relationships that could be construed as a potential conflict of interest.

## Abbreviations

ANOVA: analysis of variance
EMG: electromyogram
MIS: multiplication index of synergy
MVC: maximum voluntary contraction.
